# Supplementary material for: Methodological Aspects in Randomized Clinical Trials of Nutritional Interventions
Source: Nutrients. 2022 Jun 7;14(12):2365. doi: 10.3390/nu14122365 (PMC9227614; doi:10.3390/nu14122365)
Supplement: Supplementary file 1 [file nutrients-14-02365-s001.zip › nutrients-1741653-supplementary.pdf]

S1. Simple randomization in R.

Performing simple randomization in R is easy, and there is no need to install libraries. For this, the sample function is used. For example, suppose we want to randomize participants into two groups (A and B), and we want to randomize a sample of 50 patients. The code would be of help:

```
1. Creation of a vector with my study groups
groups <- c("A", "B")
# 2. Creation of a vector with the randomization of the groups
random <- sample(x = groups, size = 50, replace = T)
# Were:
  # x: vector on which the randomization will be performed
  # size: a non-negative integer giving the number of items to choose
  # replace: should sampling be with replacement?
# 3. randomization display
random
# 4. visualization of how many patients were assigned to each group
table(random)
```

The above function generates unequal numbers in both groups. If you intend to have groups with equal numbers of patients, you can use the blockrand function from the blockrand library. See (https://cran.r-project.org/web/packages/blockrand/index.html accessed on 3 April 2022)

S2. Stratified randomization in R.

Performing stratified randomization in R requires extra libraries outside the base package. One of them is the blockrand library (mentioned above). However, from our perspective, a more straightforward way of performing stratified randomization is to use the psych library (see: https://cran.r-project.org/web/packages/psych/psych.pdf accessed on 3 April 2022) with the block function random. Suppose we wish to randomize 100 subjects, stratifying by sex into two intervention groups (A and B). The following code statements contain the resolution for this stratification:

```
# 1. Creation of an object with stratified randomization
# the sex stratum contains two levels, 1 = female and 2 = male
# the group stratum contains two levels, 1 = A and 2 = B
random <- psych::block.random(n=100, ncond=c(sex=2,
                                groups = 2))
# 2. View the results
random
```

The second parameter can be used to change the number of strata and their levels.

S3. Randomization covariate using R.

There are some packages in R that allow covariant randomization (https://cran.r-project.org/web/packages/carat/carat.pdf#page2 accessed on 3 April 2022). However, from our point of view the Minirand function of the Minirand library allows the Minimization randomization to k treatment groups in a simpler way. To use this function several things are necessary; a matrix or a data frame of the variables that will be considered as cofactors, the treatment number, a vector with the weights of each of the covariates and the number of treatments. The detailed explanation of this function is beyond the scope of this review, but readers can check (https://cran.r-project.org/web/packages/Minirand/Minirand.pdf accessed on 3 April 2022) for more information on the matter. However, an example

adapted from Minirand CRAN website is described. In this example, we randomized 120 subjects into three interventions taking into account age, gender, obesity, and diabetes.

```
library(Minirand) #For attach Minirand library
# Preparing de data
ntrt <- 3 #Number of treatments or interventions
nsample <- 120  # Sample, number of individuals you want to randomize
trtseq <- c(1, 2, 3) # vector of a sequence of treatment groups
ratio <- c(2, 2, 1) # vector of randomization ratios for each treatment
# generate data
c1 <- sample(seq(1, 0), nsample, replace = TRUE, prob = c(0.4, 0.6))
c2 <- sample(seq(1, 0), nsample, replace = TRUE, prob = c(0.3, 0.7))
c3 <- sample(c(2, 1, 0), nsample, replace = TRUE, prob = c(0.33, 0.2, 0.5))
c4 <- sample(seq(1, 0), nsample, replace = TRUE, prob = c(0.33, 0.67))
# generate the matrix of covariate factors for the subjects
covmat <- cbind(c1, c2, c3, c4) # Combine columns in a matrix
colnames(covmat) = c("Gender", "Age", "Obesity", "Diabetes") # label of the covariates
covwt <- c(1/4, 1/4, 1/4, 1/4) #vector of weights of the covaraite factors equal weights
res <- rep(100, nsample) # result is the treatment needed from minimization method
#gernerate treatment assignment for the 1st subject
res[1] = sample(trtseq, 1, replace = TRUE, prob = ratio/sum(ratio)) f
or (j in 2:nsample)
{
# get treatment assignment sequentiall for all subjects
res[j] <- Minirand(covmat=covmat, j, covwt=covwt, ratio=ratio,
ntrt=ntrt, trtseq=trtseq, method="Range", result=res, p = 0.9)
}
trt1 <- res
#Display the number of randomized subjects at covariate factors
balance1 <- randbalance(trt1, covmat, ntrt, trtseq)
balance1
totimbal(trt = trt1, covmat = covmat, covwt = covwt, ratio = ratio, ntrt = ntrt, trtseq =
trtseq, method = "Range")
```
